# Knee Joint Biomechanics in High Flexion

**DOI:** 10.1155/2014/459408

**Published:** 2014-07-17

**Authors:** Shunji Hirokawa, Kazuo Kiguchi, Mitsugu Todo, Bharat Mody, Ashvin Thambyah

**Affiliations:** ^1^Research Center for Advanced Biomechanics, Kyushu University, 744 Motooka, Nishi-ku, Fukuoka 819-0395, Japan; ^2^Department of Mechanical Engineering, Faculty of Engineering, Kyushu University, 744 Motooka, Nishi-ku, Fukuoka 819-0395, Japan; ^3^Division of Renewable Energy Dynamics, Research Institute for Applied Mechanics, Kyushu University, Kasuga-koen, Kasuga, Fukuoka 816-8580, Japan; ^4^Welcare Hospital, Akashganga Complex, Race Course Circle, Baroda, Gujarat 390 007, India; ^5^Department of Chemical and Materials Engineering, Faculty of Engineering, University of Auckland, Symonds Street, Auckland 1010, New Zealand

A complete understanding of joint biomechanics is important in the diagnosis of joint disorders, in the quantitative assessment of treatment outcomes, and in the improvement of prosthetic devices. Yet, there is little data on high flexion activities, despite the fact that these activities are considered not only crucial to people in Asia and the Middle East, but also necessary to people in the West for improving their quality of life. Thus, we planned to invite investigators to contribute original research articles as well as review articles that will stimulate the continuing efforts to develop a thorough knowledge base for biomechanics of the knee joint during demanding activities of daily living.

To this end, the special issue has been posted online on the journal's website, in which the following articles are offered for public viewing.

“*Squatting-related tibiofemoral shear reaction forces and a biomechanical rationale for femoral component loosening*” by A. Thambyah and J. Fernandez deduces that the rapid reversal of the tibiofemoral shear force from the posterior to the anterior direction from knee extension to high flexion, respectively, may cause the femoral component loosening of high flexion implants. In this article, the results from biomechanical and finite element analyses clearly explain the relationship between the femoral loosening and frequent squatting.

“*The effect of malrotation of tibial component of total knee arthroplasty on tibial insert during high flexion using a finite element analysis*” by K. Osano et al. pertains to the finite element analysis to study the effect of malrotation of tibial component on tibial insert during squatting maneuver. Three different tibial rotations (15° internal, normal, 15° external) were set as the initial conditions for analyses. Then it was found that when the tibia was internally rotated the insert had such high stress as 160% of normal position. It was concluded that internal malrotation should be avoided as much as possible.

“*Biomechanical considerations in the design of high-flexion total knee replacements*” by C.-K. Cheng et al. is the review article in which the authors share several experiences in the previous literature for biomechanical and kinematic performance of knee prostheses under the specific demand for high flexion of knee joint. For achieving deep knee flexion, some ideas are discussed such as a rounded and convex lateral plateau and a shallower medial plateau on the tibial surface, a rounded postcam contact surface, and an asymmetric curve-on-curve cam shape.

“*Dynamic finite element analysis of mobile bearing type knee prosthesis under deep flexional motion*” by M. A. M. Anuar et al. also pertains to the finite element analysis to distinguish the mechanical performances between mobile bearing and fixed bearing posterior stabilized knee prostheses. Then this study shows the decomposition of multidirectional motion to unidirectional kinematics of femoral-insert and insert-tray articulating interfaces in mobile bearing TKA in maintaining lower stress at tibial condylar.

“*Modelling and analysis on biomechanical dynamic characteristics of knee flexion movement under squatting*” by J. Wang et al. represents the results from the synchronal measurements of both kinematics and contact stresses, respectively, for the tibiofemoral and patellofemoral joints. Dynamic finite element analysis and in vitro experiment were carried out for the range from full extension to squatting of the knee. Comparative study between their results and the literature data is comprehensive.

We hope that the above articles are of interest to the reader and more articles would be incorporated in this special issue.

*Shunji Hirokawa* *Shunji Hirokawa* *Kazuo Kiguchi* *Kazuo Kiguchi* *Mitsugu Todo* *Mitsugu Todo* *Bharat Mody* *Bharat Mody* *Ashvin Thambyah* *Ashvin Thambyah*

